# German Universities and English Students

**Published:** 1890-10-25

**Authors:** 


### The Hospital

A Weekly Journal of

Science, Medicine, Nursing, and Philanthropy.

*For Hospitals, Asylums, Medical Practitioners, Students, Nurses, Families, Parishes, Congregations, and the Charitable Public.*

Vol. IX.—No. 213.] [October 25, 1890.

### German Universities and English Students.

The subject of continental, and especially of German universities, is one which is growing almost daily of more importance to students of medicine, and especially to those who have recently qualified. A few years ago it was a very exceptional thing for a man after completing his curriculum at some British school to go for a few months to study in a foreign university. Of late years, however, modern facilities for travelling, together with the more general knowledge of the scientific advancement of many of the foreign universities, have tended to encourage the young practitioner to extend his sphere of medical and scientific education, and not immediately to settle down to practice content to follow implicitly the teaching of some one master.

The first point we will briefly discuss is, what may be considered the advantages of study at a German university? In writing on this subject we shall, of course, conclude that the student has completed his home curriculum, and, in fact, has become qualified. It is admitted by all who have had practical experience of the subject that with regard to pure science the Germans are a long way ahead of Englishmen. Unpatriotic, as this view must appear to many, it is nevertheless true, and the sooner we, as a nation, realise and endeavour to remedy this great blot upon our educational departments, the better. The advantages to be gained by a few months' residence in one of the great foreign centres of education are very great. This is especially true of the departments of medical science devoted to pathology and physiology, which may be considered purely scientific as distinguished from the more practical ones. With regard to pathology, the German professor stands out prominently with his deeply imbued spirit of scientific research. The result of this has been the great increase of accommodation, and with this the accumulation of material for pathological study. Thus, in all the schools the pathological department forms a most important part of the building, consisting of several laboratories built and kept solely for this branch of medicine, and not used for any other. The professor, also, devotes the whole of his time to teaching, not because it brings him in so much a-year, but because he has selected the subject in which he is most interested, and the one to which he intends devoting his life; both the professor and his assistants spend the whole day in the laboratory with the students, and so bring themselves into constant personal contact with them. The same might be said concerning physiology, which subject also forms a very extensive section of the laboratory departments.

Passing on to the more practical spheres of medicine proper, namely, medicine and surgery, much may be learnt in both of these departments; in the former the more theoretical methods generally in use with regard to the treatment of disease, and in the latter the much greater boldness of the German surgeon as compared with the English. But, perhaps of all the departments of the purely professional subjects the greatest gain will be derived from the study of some speciality, such for instance as diseases of the skin, eye, or throat. Germans are disposed to specialise even more than the English; that is to say, when they take a special subject they pursue it to its fullest scientific basis, so that in most of the Universities all the special subjects form large and most complete departments, each quite self-contained, and yet all under the same general organisation, and the same central controlling body. In this way all the specialities get as much attention attached to them as the more general branches of medicine and surgery. In many of the schools the material to be obtained in the special departments is simply enormous, and every possible facility is given to the foreigner to make himself master of his subject. In some of the Universities, especially so in Vienna, nearly all of the courses are now arranged for the convenience of the foreigners, rather than for the use of the native students. This is chiefly due to the great numbers of aliens at present working there, especially American doctors, of whom we have heard it stated that there are usually between one and two hundred studying at Vienna University, and that some of the courses have even been conducted in English to please the strangers.

The most important questions that arise in the mind of a student desirous of working abroad are where to go, and out of all the numerous Universities which is the best? In the first place the language must be learnt, or at least sufficient of it to enable one to carry on ordinary conversation. With no previous knowledge of the tongue it is of very little use to go direct to a University town, or in fact to a large town at all. Upon arriving at a University the student at once finds out some of his countrymen, many of whom have a wonderful bias in favour of their native tongue, even if they can speak something of the other one, and with these he speaks English and English only. At one University there is, or was, a large club for the reception of members of the English speaking nations, the first rule of which provided that for every word of German spoken by any of its members, a fine was to be imposed. This club was chiefly patronised

Army Medical Library Washington, D. C. U. S.

by young men sent to Germany by their parents to cram for the army, with the special object of learning German. By far the best plan is at first to live with a German family, right away in some part of the country, where the stimulus afforded by the desire to get away to more active scenes of life will encourage the rapid acquirement of sufficient words of the language for daily use. When the student has accomplished the art of ordinary conversation then he will do well to seek a university town. Which city he should go to depends very much upon what he especially wishes to work at, one place being very good in some branches, and another in others. But the chief aim for anyone, not very well up in the language, should be to go where there are fewest of his countrymen, a place more difficult to find every year, as now so many more students go for a time to work abroad, and there are but few towns left that are not inundated with Americans and others speaking English. So also the difficulty is becoming greater, because so many Germans now speak a little English, and are never shy of using this little; in this respect showing a marked contrast to the Englishman, who will never speak a word of German if he can find anyone who understands a little English.

Life in a German university is essentially different to ours; lectures commence comparatively early; in the summer in many universities the clinic commences at six a.m. daily, and in the winter it begins usually at eight o'clock in the morning. Thus the students work more in the daytime and less at night than we do; in fact, unless just before an examination, or in very exceptional cases, there is no reading done in the evenings, which are spent by the students in various cafés, drinking a very light form of beer or playing billiards. Genuine outdoor sports are almost unknown; football or cricket is but very rarely seen, but rowing of a kind is becoming much more general in those towns where there is a river.

In concluding an article upon German Universities as a means of education for an English medical man, we must say a few words upon education in general. Not many years ago a little knowledge of Latin and Greek grammar, a few disconnected dates of comparatively useless events in history, together with a smattering of ecclesiastical dogma, were considered to form the essentials in the education of a gentleman. Now much is being written on the subject, and some few try to revive the flickering flame of the classical basis of education; we believe, however, that the day is not far distant when, instead of a knowledge of the ancients being essential, personal acquaintance with the people at present living will be considered to form the necessary part of the education of a gentleman, rather than a few isolated facts of the lives of pre scientific generations of men.

As useful and practical men of medicine, our chief object in life should be the study of man. To do this fully we must live in the great centres of civilization, and whilst there live with, and endeavour to interest ourselves in, those things which most concern the people amongst whom we are living, for in this way, and in this way only, shall we get an insight into the manners and customs of other peoples. The student's mind will be wonderfully expanded by such dealings with men, and he will be able to understand the various ways different individuals look at things, and so gradually learn to act in all circumstances with wise circumspection and sound judgment.

